# Correlative analysis of metallomic gene expression and metal ion content within the mouse hippocampus

**DOI:** 10.1093/mtomcs/mfaf009

**Published:** 2025-04-02

**Authors:** Somayra S A Mamsa, Gaewyn Ellison, Julia Koehn, Keea Inder-Smith, Cameron W Evans, Ross M Graham, Daryl L Howard, Mark J Hackett

**Affiliations:** Curtin Medical Research Institute, Curtin University, Bentley, WA 6102, Australia; School of Molecular Sciences, The University of Western Australia, Crawley, WA 6009, Australia; Curtin Medical Research Institute, Curtin University, Bentley, WA 6102, Australia; School of Molecular and Life Sciences, Faculty of Science and Engineering, Curtin University, Bentley, WA 6102, Australia; Curtin Medical Research Institute, Curtin University, Bentley, WA 6102, Australia; Curtin Medical Research Institute, Curtin University, Bentley, WA 6102, Australia; Curtin Medical School, Curtin University, Perth, WA 6102, Australia; School of Molecular Sciences, The University of Western Australia, Crawley, WA 6009, Australia; Curtin Medical Research Institute, Curtin University, Bentley, WA 6102, Australia; Curtin Medical School, Curtin University, Perth, WA 6102, Australia; Australian Synchrotron, ANSTO, Clayton, VIC 3168, Australia; Curtin Medical Research Institute, Curtin University, Bentley, WA 6102, Australia; School of Molecular and Life Sciences, Faculty of Science and Engineering, Curtin University, Bentley, WA 6102, Australia

## Abstract

Brain metal homeostasis is essential for healthy neurological function, and disturbed brain metal homeostasis has deleterious consequences for neurodevelopment or cognitive outcome following injury or during disease. Specific regions of the brain (e.g. the hippocampus and subregions within) are known to be enriched with transition metals (i.e. ions of iron, copper, and zinc). Neither the physiological need for localized enrichment, nor the mechanisms driving the enrichment, however, are well understood. In this study we have applied a multimodal template, incorporating elemental mapping using X-ray fluorescence microscopy with spatial transcriptomics, to help reveal a molecular basis for metallomic heterogeneity across key subregions of the hippocampus. Our results reveal that significant differences in iron, zinc, and copper enrichment are associated with regional enrichment of specific transcripts related to metal transport, metal storage, and metal regulatory proteins. In addition to providing novel biological insight into the neurometallomic profile of the hippocampus, this study also provides an important template for others to integrate transcriptomics into multimodal workflows investigating the neurometallome.

## Graphical Abstract

**Graphical Abstract:**
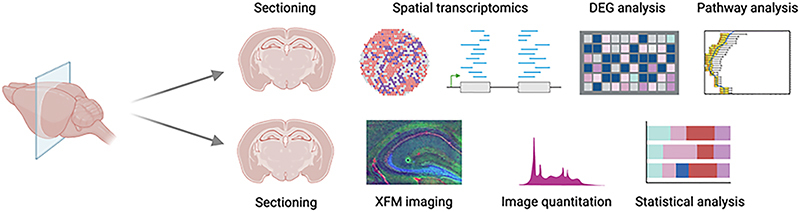

## Introduction

Brain metal homeostasis is essential for healthy neurological function. In particular, both insufficient and excess levels of iron, zinc, and copper are associated with adverse effects on neuronal development and survival. However, the distribution of these metals across the brain is highly heterogeneous between anatomical subregions. In the hippocampus, a relatively high level of iron is found in the cornu ammonis sector 1 (CA1) pyramidal cell layer [1, 2], where it has a key role in long-term potentiation and memory formation [3, 4]. Iron deficiency adversely affects neurological development and cognition [5], while accumulation of excess iron may induce oxidative stress, protein aggregation and neurodegeneration [6], and constitutes a pathological hallmark of Alzheimer's disease [7]. Iron deficiency and accumulation may occur concomitantly, with the process of iron enrichment in certain cells or brain regions potentially creating deficiency in other regions or cells, as recently reviewed [8]. The hippocampus also contains a substantial quantity of zinc largely confined to the mossy fibre layer, a region comprised of axons emerging from the dentate granule cells of the dentate gyrus (DG), passing through the hilus of the DG, and entering the stratum lucidum of the cornu ammonis sector 3 (CA3). Zinc ions bind to an estimated 10% of the proteome for either structural stability, or as a cofactor for enzymatic activity [9]. In the brain, the majority of zinc is bound to proteins, with a lower proportion stored as mobile zinc in the synaptic vesicles of glutamatergic nerve terminals to modulate neurotransmission [10]. Abnormally high and low levels of zinc have both been implicated in neurodegenerative diseases such as Alzheimer's disease [11]. Copper is an essential cofactor for proteins involved in mitochondrial function, neurotransmitter synthesis and redox homeostasis throughout the brain, and is also believed to negatively regulate neurogenesis [12]. In the developed brain, high amounts of copper are therefore found in the subventricular zone (SVZ) of the lateral ventricles [13, 14], which serves as the main neurogenic niche of the brain.

Despite the established importance of metal ions to brain function, in particular to hippocampal function, the molecular basis for metal enrichment in specific hippocampal subfields (e.g. CA1, CA3, DG) and surrounding structures (SVZ) is not well characterized. It is important to fill this gap, as knowledge of the underlying molecular mechanisms that result in metal enrichment within specific brain regions or cell populations is likely to be a key step in both understanding the pathways through which metal dyshomeostasis occurs during neurodegenerative disease, and identifying potentially therapeutic strategies. Therefore, we aimed to characterize transcriptomic profiles associated with differential iron, zinc, and copper enrichment in various subregions of the brain, particularly the CA1, CA3, and DG of the hippocampus, the cerebral cortex (CTX), corpus callosum (CC), and the SVZ of lateral ventricles. As the SVZ is a heterogeneous brain region, our analyses in this study were confined to the ependymal cell layer of the lateral ventricles, a specialized layer of glial cells (referred to herein as the ventricle wall, VW). While traditional approaches to studying gene expression require the extraction of transcripts from homogenized brain tissue, the regional specialization of the brain necessitates approaches which conserve anatomical information. Spatial transcriptomics allows the simultaneous detection and quantification of thousands of transcripts while preserving their spatial context. Therefore, we applied for the first time (to the best of our knowledge) a multimodal approach combining direct elemental mapping of brain tissue sections through X-ray fluorescence microscopy (XFM) with *in situ* spatial transcriptomics analysis of serial sections collected from the same brain samples, to associate metallomic and transcriptomic profiles within specific hippocampal subregions. The results highlight key transcriptomic signatures relating to metal ion transporters, storage proteins, and metallo-proteins that are enriched in association with the metal ions themselves. In addition to this fundamental characterization of the neurobiology of the hippocampus, this study serves as a template for others to adapt multimodal elemental mapping with spatial transcriptomics.

## Methods

### Tissue collection

All animal procedures were approved by the Animal Ethics Committee at Curtin University under approval number ARE2022-5. Wild-type C57BL6J mice aged 7 months (*n *= 5) were maintained on a standard chow diet with access to water *ad libitum* and housed with a 12 h day/night light cycle. Prior to tissue collection, mice were anaesthetized using isoflurane, and euthanized by exsanguination followed by cardiac removal. Brains were collected and cut mid-sagittally into hemispheres, flash-frozen in liquid nitrogen-cooled isopentane, and stored at −80°C until sectioning. Tissue was mounted onto the sectioning chuck using OCT medium and cryosectioned at 10 µm, with coronal sections collected directly onto glass slides for immunofluorescence and spatial transcriptomics analyses, and onto silicon nitride membranes for XFM. Tissue sections for spatial transcriptomics were stored at −80°C for 7 days before being thawed and immediately immersion fixed in 10% formalin.

### Immunofluorescence

The SVZ can be identified by high density of ependymal cells positive for glial fibrillary acidic protein (GFAP) along the lateral VW. Therefore, GFAP positive immunofluorescence was used to confirm the location of the ependymal cells in VW in this study. Tissue sections were incubated with Nanostring Blocking Buffer W (NanoString) for 30 min in the dark, followed by incubation with an Alexa Fluor 647-conjugated anti-GFAP antibody (1:200 dilution) and the nuclear stain CYTO83 (1 μM, Thermo Fisher S11364) for 1 h in the dark, then washed three times with PBS.

### Spatial transcriptomics data acquisition

Spatial transcriptomics was performed using the NanoString GeoMx Digital Spatial Profiler (DSP) (NanoString). Targets were exposed by heat-induced target retrieval (100°C for 15 min) using Tris-EDTA buffer (eBiosciences 00-4956-58), and proteinase K digestion (Thermo Fisher AM2548, 0.1 ug/ml final concentration, 37°C for 15 min). Target detection was performed with the whole transcriptome atlas from NanoString. The nuclear stain CYTO83 was used for ROI (Region of Interest) selection, with ROIs for the CA1, CA3, DG, CC, CTX, and VW defined manually based on anatomical structure. It has been established in our past work [1], and the work of others [15, 16], that there is variation in metal content within specific hippocampal subregions, such as a prominent lateral to medial trend in Fe content across the CA1 and CA3 pyramidal cell layers. Therefore, to try and minimize data variation introduced by these trends, ROIs were kept as small as possible (while still maintaining sufficient material for signal amplification, described below). As the ROIs were drawn by hand, it should be noted that there is likely to be a small amount of ‘bleed through’ from adjacent tissue, particularly for narrow ROIs, corresponding to neuronal layers (e.g. CA1, CA3, and DG). All ROIs were between 30 000 and 50 000 μm^2^ and contained between 200 and 500 cells. Illumina sequencing was performed by the Australian Genome Research Facility, and FastQ files were converted into DCC (Data Coordination Centre) files using the GeoMx Pipeline Software (NanoString).

Selection of ROI size is an important consideration for spatial transcriptomics, as although the RNA tags can be measured with a precision defined by the diffraction limit of UV light (∼0.2 µm), the signal amplification still requires a minimum amount of tissue material. The minimum amount of material equates to ∼100 cells, which is an effective area of 200 µm × 100 µm (or 20 000 µm^2^, assuming a single brain cell is ∼20 µm × 10 µm). Due to the high cost of spatial transcriptomics, we used conservative ROIs of size 30 000 µm^2^ (200 µm × 100 µm) in this study, to avoid loss of data due to insufficient cell material for signal amplification.

### Spatial transcriptomics data processing

All initial data processing of spatial transcriptomics data was performed using the NanoString GeoMx DSP Data Centre analysis platform. Quality control was performed to remove reads below the limit of quantitation (LOQ), defined as the negative probe geomean multiplied by the geometric standard deviation of the negative probe. Remaining data above the LOQ were normalized through upper quartile (Q3) normalization, and background correction was performed using negative probe subtraction.

### Differential gene expression analysis

**Figure 1.**
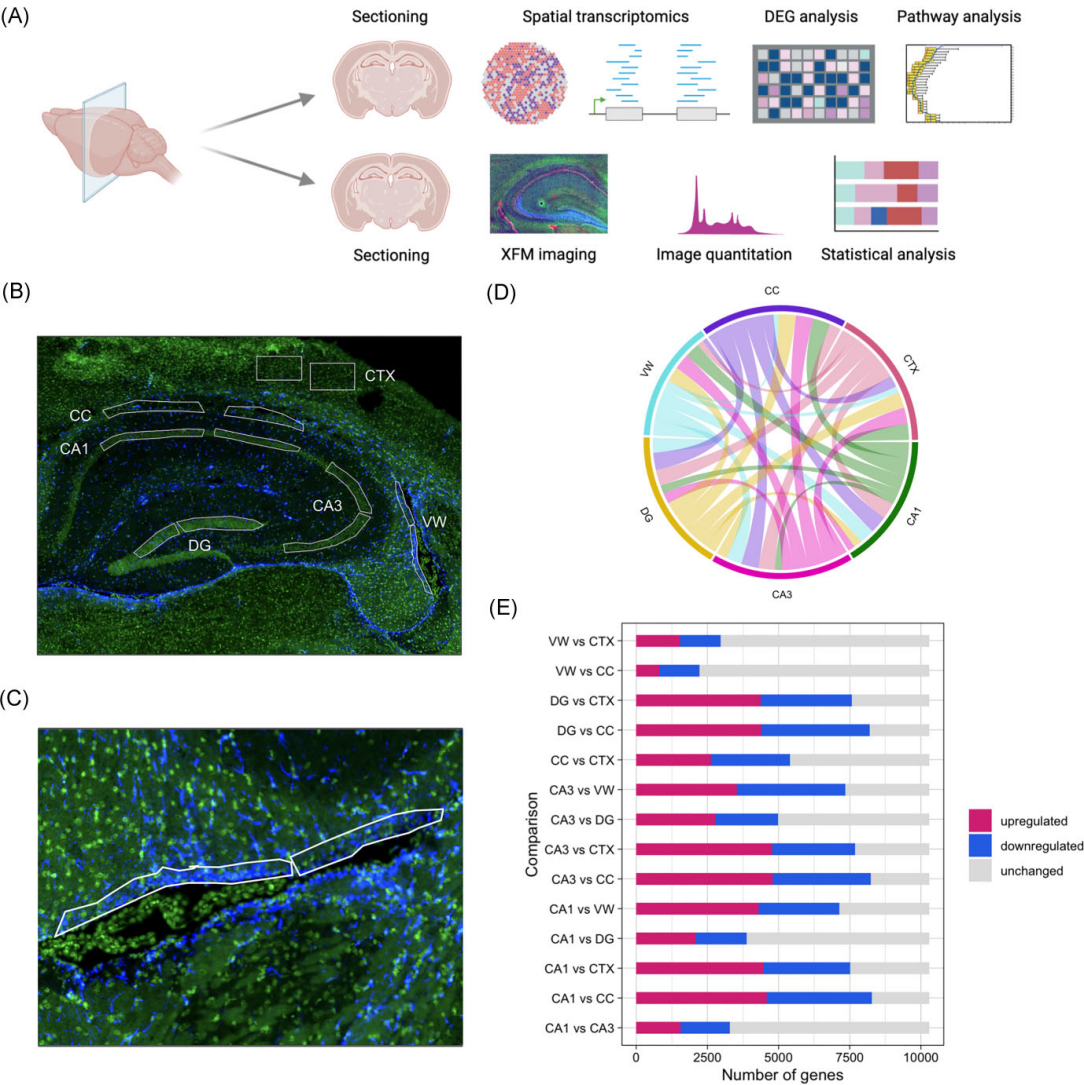
Overview of analysis workflow and spatial transcriptomic comparisons. (a) Schematic of workflow. (b) Representative cyto83 DNA stain and GFAP-immunofluorescence image with annotations for anatomical subregions used in spatial transcriptomics. green: cyto83 DNA stain. blue: anti-gfap antibody. (c) Representative immunofluorescence image of GFAP antigenicity (blue) along the VW. (d) Chord diagram weighted by total number of differentially expressed genes (*P* < .05) between subregions. (e) Number of significantly (*P* < .05) upregulated, downregulated, and not significantly different genes between subregions.

Differential gene expression analysis was performed using the NanoString GeoMx DSP platform. ROIs were annotated by anatomical subregion, and gene expression levels were compared between subregions from five mice (*n* = 5 biological replicates) using a paired *t*-test, with *P*-values adjusted through Benjamini-Hochberg correction to control for the false discovery rate. Genes were considered differentially expressed when adjusted *P* < .05. At the time of data collection, two technical replicates were collected for each subregion, as shown in Fig. 1. Technical replicates were averaged together to yield a single value per animal for statistical analyses.

### Pathway enrichment analysis

All pathway enrichment analyses were performed using R v4.3.1 in RStudio v2023.12.0 + 369. Gene names for differentially expressed gene (DEG) sets were converted to Entrez IDs through *dplyr* v1.0.8 and *AnnotationDbi* v1.56.2 using the *Org.Mm.eg.db* v3.14.0 *Mus musculus* annotation database. Pathway enrichment analysis using Gene Ontology (GO) mapping was performed through *pathfindR* v1.6.3 [17], using gene set data for *Mus musculus* from the Reactome database [18]. *P*-values were adjusted using Benjamini-Hochberg correction, and enriched pathways with adjusted *P* < .05 were considered statistically significant.

### X-ray fluorescence microscopy

Metal mapping was undertaken using XFM, at the XFM beamline of the Australian Synchrotron (Australian Nuclear Science and Technology Organisation) [19]. Metal maps were collected using a monochromatic incident beam of 15.8 keV, focused to a 1 µm (1-sigma) spot with a Kirkpatrick–Baez mirror pair, with data collected using a pixel size of 1 µm and a dwell time of 1 ms (X-ray flux was calculated to be 5.3 × 10^10^ photons/s upstream of KB mirrors, and estimated to be 3 × 10^10^ photons/s in the focused X-ray beam spot on the sample). The full X-ray emission spectrum from the sample was collected using a 4-element Vortex Si-drift detector. Elemental foils (Micromatter, Canada) were scanned in the same geometry as the samples (backscatter geometry) and used as references for elemental quantification, as per past publications [1, 2].

### Image processing

Elemental maps were reconstructed from the emission spectra using GeoPIXE v6.6j (Commonwealth Scientific and Industrial Research Organisation, Australia), which implements a linear transformation matrix for spectral deconvolution. Data were exported as TIFF files of quantitative per-pixel elemental area density (ng cm^−2^), and the images were then processed in FIJI v2.9.0 using the native function for outlier removal with a radius of 2.0 pixels and threshold value of 50. ROIs were manually selected based on anatomical subregions, and quantitative data was measured and extracted from each ROI. Mean concentrations of Fe, Zn, and Cu measured from the blank substrate were subtracted from the mean concentration values for each region to give the final concentration of each element.

### Statistical analysis of XFM data

Statistical analyses for quantitative XFM data were performed in R v4.3.1 using *rstatix* v0.7.2. For comparison of subregions, elemental data from four mice (*n* = 4) were analysed for each ROI using a one-way ANOVA with Tukey's post-hoc test. For comparison of the medial vs lateral CA1, a one-sided *t*-test was used. There was one fewer animal replicate in the XFM dataset (*n* = 4), compared to the transcriptomic dataset (*n* = 5), due to sample damage during transport.

## Results & discussion

### Brain subregions exhibit distinct transcriptomic and metallomic profiles

A multimodal approach combining spatial transcriptomics and XFM elemental mapping was applied to serial sections from the same tissue samples, as outlined in Fig. 1a. Using spatial transcriptomics, 10 299 genes were detected above the LOQ. Comparison of DEGs between manually defined subregions for the CA1, CA3, DG, CC, CTX, and VW (Fig. 1b) revealed distinct transcriptomic profiles. The most extensive differences were between each of the hippocampal subfields and the CC, with 8246 DEGs in the CA1, 8231 DEGs in the CA3, and 8199 DEGs in the DG (Fig. 1e), suggesting the hippocampus overall exhibits a vastly different transcriptome from the CC. Fewer differences were observed when comparing the nonhippocampal subregions (e.g. VW and CTX) to the CC (Fig. 1e).

**Figure 2.**
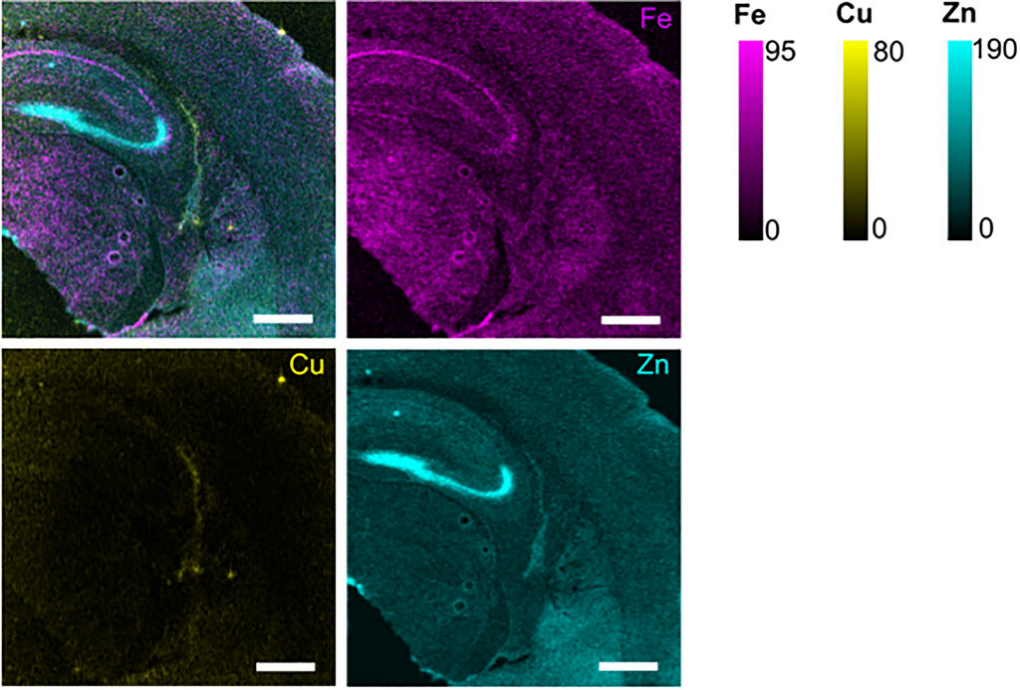
XFM elemental mapping showing merged Fe/Cu/Zn overlay, and individual Fe, Zn, and Cu maps in the hippocampus and adjacent regions. Physical scale bar = 500 µm. Units for elemental areal density scale are ng cm^−2^.

Given the significantly different transcriptomic profiles between hippocampal subfields observed in this study, associated elemental differences were also expected across the regions analysed. Indeed, qualitative visual inspection of XFM images (Fig. 2) demonstrates the trends of metal ion distribution across the hippocampus subregions. Specifically, elemental maps indicated clear enrichment of Fe in the CA1 pyramidal neuron layer, enrichment of Zn in the ‘mossy fibres’ of the CA3 and DG subfields (tissue layer containing synapses from CA3 and DG neurons), and enrichment of Cu in the VW (Fig. 2). The specific association between metal ion content and transcriptomic signatures within individual subfields is described below.

### Transcriptomic signatures of iron enrichment in the CA1 subfield

**Figure 3.**
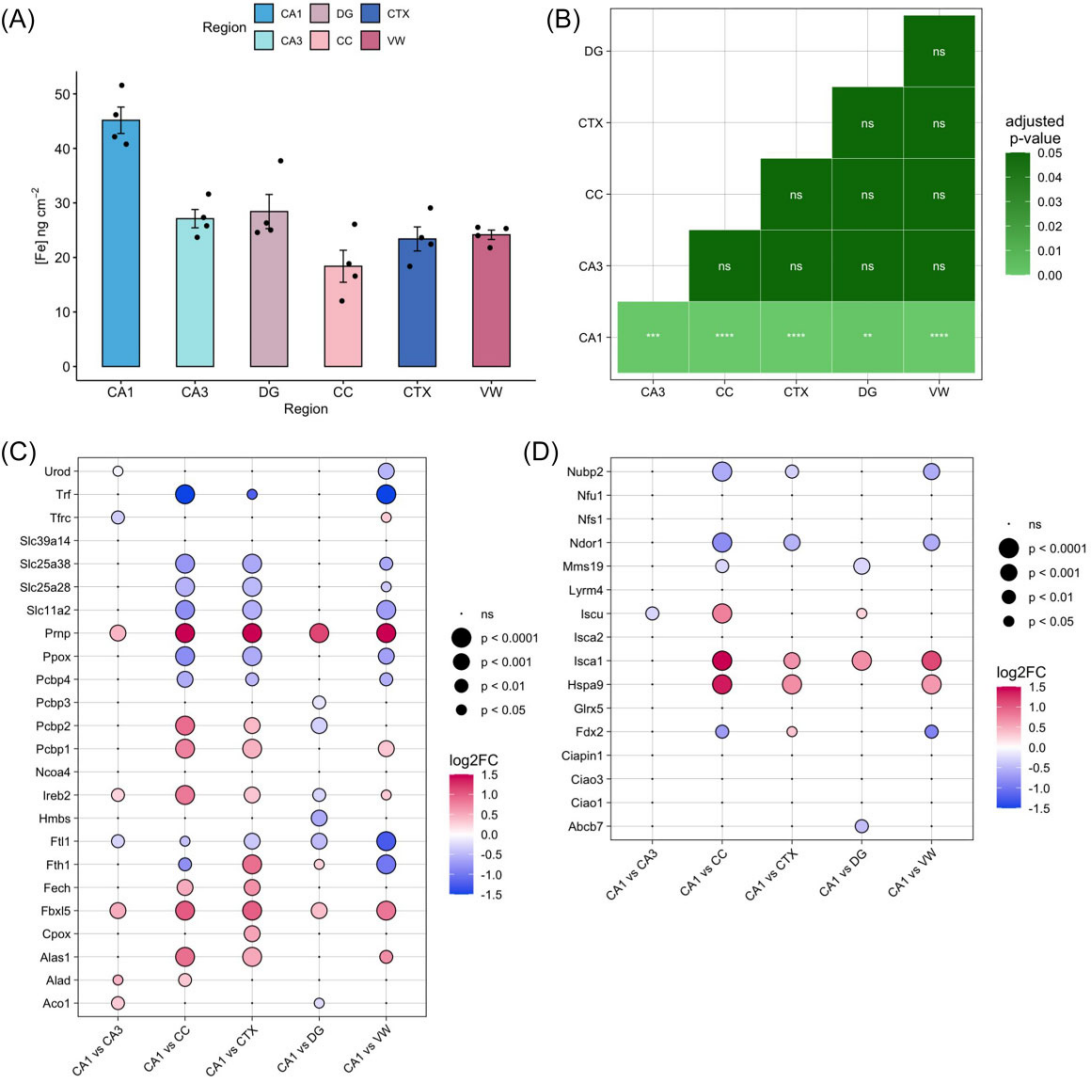
Iron abundance and expression of associated transcripts in the brain: (a) Elemental concentration of iron ± SE in the CA1, CA3, DG, CC, CTX, and VW. (b) Significance of pairwise comparisons of iron content by Tukey's post-hoc test are shown between regions. Differential expression of genes relating to iron homeostasis in the CA1: (c) expression of genes related to iron transport, metabolism and storage. (d) Expression of genes related to iron-sulphur cluster assembly. CA1 = cornu ammonis sector 1, CA3 = cornu ammonis sector 3, DG = dentate gyrus, CTX = cortex, CC = corpus callosum, VW = ventricle wall. *P < .05; **P < .01; ***P < .001; ^****^P < .0001.

Quantitative analysis of XFM data highlighted significantly higher levels of iron in the CA1 when compared to the CA3, DG, CC, CTX, and VW (Fig. 3a), consistent with a prior XFM study of younger mice [1]. Comparing the remaining subregions with each other did not reveal any differences in iron concentration (Fig. 3a). The enrichment of iron in the CA1 is consistent with functional studies indicating that iron is required for the development and maturation of CA1 pyramidal neurons [20], long-term potentiation in developed CA1 pyramidal neurons [3], and spatial memory acquisition [4].

A transcriptomic basis for iron enrichment in the CA1 was next investigated. Several key trends were observed in the expression of genes relating to iron transport and metabolism when comparing the CA1 to the CA3, CC, CTX, DG, and VW (Fig. 3c). Notably, expression of *Prnp*, encoding the prion protein (PrP), was consistently higher in the CA1 than in the remaining subregions (Fig. 3c). PrP is a key mediator of iron uptake and transport in the brain; *in vitro* studies indicate PrP increases total cellular iron by increasing both the pool of labile iron and iron stored in cytosolic ferritin [21], and mice lacking *Prnp* show a phenotype of relative iron deficiency in the brain [22]. The upregulation of *Prnp* in the CA1 observed here suggests that the *Prnp* protein may be a mediator of increased basal iron content in the CA1 subfield. This finding would be consistent with a past study indicating *Prnp* knockout mice contain less brain Fe than corresponding wildtype mice [23].

The remaining iron transporter proteins were not conclusively shown to be upregulated in the CA1. A slight decrease in CA1 expression of *Tfrc*, encoding the canonical iron uptake transporter, transferrin receptor (TFRC), was observed in comparison to the CA3. However, the level of *Tfrc* was consistent between the CA1 and the DG, CC, and CTX, and slightly higher than that of the VW (Fig. 3c). *Slc40a1*, encoding the iron exporter ferroportin-1 (FPN1), and *Hamp*, encoding hepcidin, a key regulator of iron homeostasis, were not detected in this study. Expression of *Slc11a2*, which encodes the metal uptake transporter DMT1, was not significantly different in the CA1 when compared to the CA3 or DG. This suggests expression of DMT1 levels are relatively consistent across the hippocampus. Interestingly, however, levels of *Slc11a2* were higher in the CTX, CC, and VW than in the CA1, which may be attributed to other roles of DMT1 in the brain, such as in maturation of oligodendrocyte precursor cells and myelination [24]. DMT1 can also transport metals other than Fe, such as Mn, Cu, and Zn, although with differing affinities [25, 26], which may also account for the higher levels of *Slc11a2* in the CTX, CC, and VW relative to CA1.

The transcriptomic signature of the CA1 broadly reflected increased expression of transcripts involved in regulating intracellular iron homeostasis, concordant with increased iron content detected through XFM. In particular, upregulation of both *Pcbp1* and *Pcbp2*, which encode the iron chaperone proteins PCBP1 and PCBP2, respectively, was observed in the CA1 compared to the CC and CTX. Importantly, PCBP1 delivers iron to ferritin [27], and PCBP2 facilitates iron trafficking into and out of the cytosol via interactions with DMT1 and FPN1 [28]. *Fbxl5*, which encodes the F box and leucine-rich repeat protein 5 (FBXL5), a key regulator of intracellular iron concentration [29], was also upregulated in the CA1 compared to all other subregions (Fig. 3a), while *Herc2*, encoding the HERC2 protein, was upregulated in the CA1 compared to the CC, CTX, and VW. HERC2 modulates iron levels by regulating the degradation of the FBXL5 protein. Depletion of *Herc2* leads to increased FBXL5 stabilization, which results in decreased intracellular levels of iron [30]. Thus, it is possible that increased *Herc2* expression may lead to a decrease in the protein level of FBXL5, resulting in iron accumulation.

Post-transcriptionally, cellular responses to iron are largely controlled by the iron regulatory proteins, IRP1 and IRP2, encoded by *Aco2* and *Ireb2*, respectively. Both IRPs bind to iron responsive elements in the untranslated regions of transcripts involved in iron homeostasis, regulating their stability and subsequent translation. IRP2 constitutes the dominant iron regulatory protein in mammals, and has a key role in post-transcriptional regulation of iron metabolism in the central nervous system [31]. Here, a higher level of *Aco2* was observed in the CA1 compared to the CA3, while *Ireb2* was significantly upregulated in the CA1 compared to all other subregions except the DG (Fig. 3b), which may indicate a higher level of iron regulatory activity in the CA1.

The CA1 did not show increased levels of transcripts encoding iron storage proteins. This is not necessarily surprising as ferritin regulation occurs post-transcriptionally. Nevertheless, *Ftl1*, which encodes the ferritin light chain protein, was downregulated in the CA1 compared to other brain subregions, while *Fth1*, encoding ferritin heavy chain, was upregulated in the CA1 only in comparison to the CTX and DG, and downregulated compared to the CC and VW. These findings may suggest that iron storage does not occur to a large extent within CA1 pyramidal neurons, with localized iron supply instead managed by surrounding glial cells such as astrocytes, which is consistent with observations of others [32, 33], and warrants further investigation.

Several transcripts relating to heme biosynthesis and degradation were also upregulated in the CA1 (Fig. 3c), including *Fech*, encoding ferrochelatase, which localizes to neurons and astrocytes [34], *Cpox*, encoding coproporphyrinogen III oxidase; *Alad*, encoding delta-aminolevulinate dehydratase; and *Alas1*, encoding 5'-aminolevulinate synthase 1. Interestingly, although perhaps not surprisingly, marked differences in the expression of multiple transcripts relating to iron-sulphur cluster assembly were observed in the CA1 region relative to CC, CTX, and VW (Fig. 3d).

### Zinc transporters are upregulated in the CA3 and dentate gyrus

**Figure 4.**
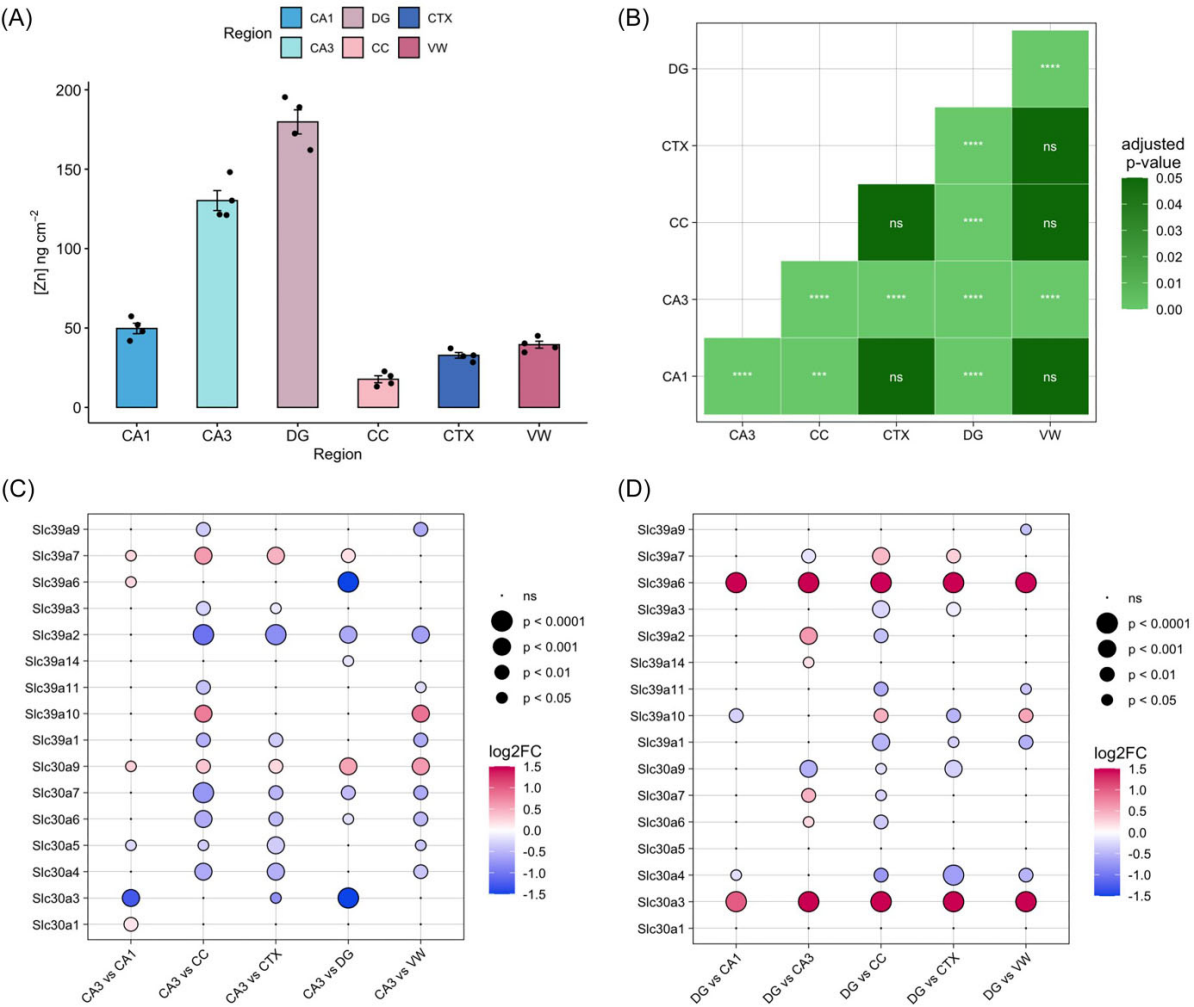
Zinc abundance and expression of associated transcripts in the brain. (a) Elemental concentration of zinc ± SE in the CA1, CA3, DG, CC, CTX, and VW. (b) Significance of pairwise comparisons by Tukey's post-hoc test are shown between regions. Differential expression of transcripts encoding ZIP and ZnT zinc transporters: (c) differential expression in subregions relative to CA3. (d) Differential expression in subregions relative to DG. CA1 = cornu ammonis sector 1, CA3 = cornu ammonis sector 3, DG = dentate gyrus, CTX = cortex, CC = corpus callosum, VW = ventricle wall. *P < .05; **P < .01; ***P < .001; ^****^P < .0001.

Analysis of XFM data showed marked enrichment of zinc in both the CA3 and DG subfields that contain ‘mossy fibres’ [35], which are Zn-enriched synaptic terminals from CA3 and DG neurons (axon from DG granule neuron that forms a synapse with CA3 pyramidal neuron dendrite). Zinc was significantly more abundant in the CA3 when compared to the CA1, CC, CTX, and VW, while the DG contained the highest concentration of zinc across all subregions, including the CA3 (Fig. 4a). These findings are consistent with elemental mapping studies [1, 36] as well as histochemical imaging [37], which has shown significant enrichment of zinc within the mossy fibres, which span the CA3 and DG subfields.

Similar to Fe, transcriptomic analysis provided a convincing molecular explanation for the localized zinc enrichment across the CA3 and DG hippocampal subfields. In the adult rodent brain, zinc homeostasis is maintained through two major families of zinc transporters: the ZnT family, encoded by *Slc30a1* to *Slc30a10*, and the ZIP family, encoded by *Slc39a1* to *Slc39a14* [38]. Here, transcriptomic profiles of zinc transporter expression within the CA3 and DG were found to differ substantially from the remaining subregions. Both the CA3 and DG showed upregulation of *Slc30a10* compared to the VW, upregulation of *Slc39a7* compared to the CTX and CC, and upregulation of *Slc39a6* compared to the CA1 (Fig. 4c, d). Further differences specific to the CA3 and DG were also observed. The CA3 showed consistent upregulation of *Slc39a7* and *Slc30a9* in comparison to all other subregions (Fig. 4c). *Slc39a7* and *Slc30a9* encode ZIP7 and ZnT9, respectively, which are essential for maintaining intracellular zinc homeostasis [39, 40]. Additionally, the CA3 showed higher expression of *Slc39a10* when compared to both the CC and the VW. *Slc39a10* encodes ZIP10, a key zinc transporter highly expressed in the brain and considered essential for neuronal zinc homeostasis [41].

In the DG, significant and consistent upregulation of *Slc30a3* and *Slc39a6* was observed in comparison to all other subregions, including the CA3 (Fig. 4d). *Slc30a3* and *Slc39a6* encode the zinc exporter ZnT3 and zinc importer ZIP6.The DG also exhibited higher expression of several zinc transporters when compared to the CC, specifically *Slc30a6, Slc30a7, Slc39a14*, and *Slc39a2*, encoding ZnT6, ZnT7, ZIP14, and ZIP2, respectively. The ZIP proteins are present on the plasma membrane [42], whilst the ZNT proteins are present in intracellular vesicles, including synaptic vesicles, and redistribute zinc from cytosol to vesicles [43], suggesting a molecular basis for the high abundance of zinc observed in the DG through XFM.

**Figure 5.**
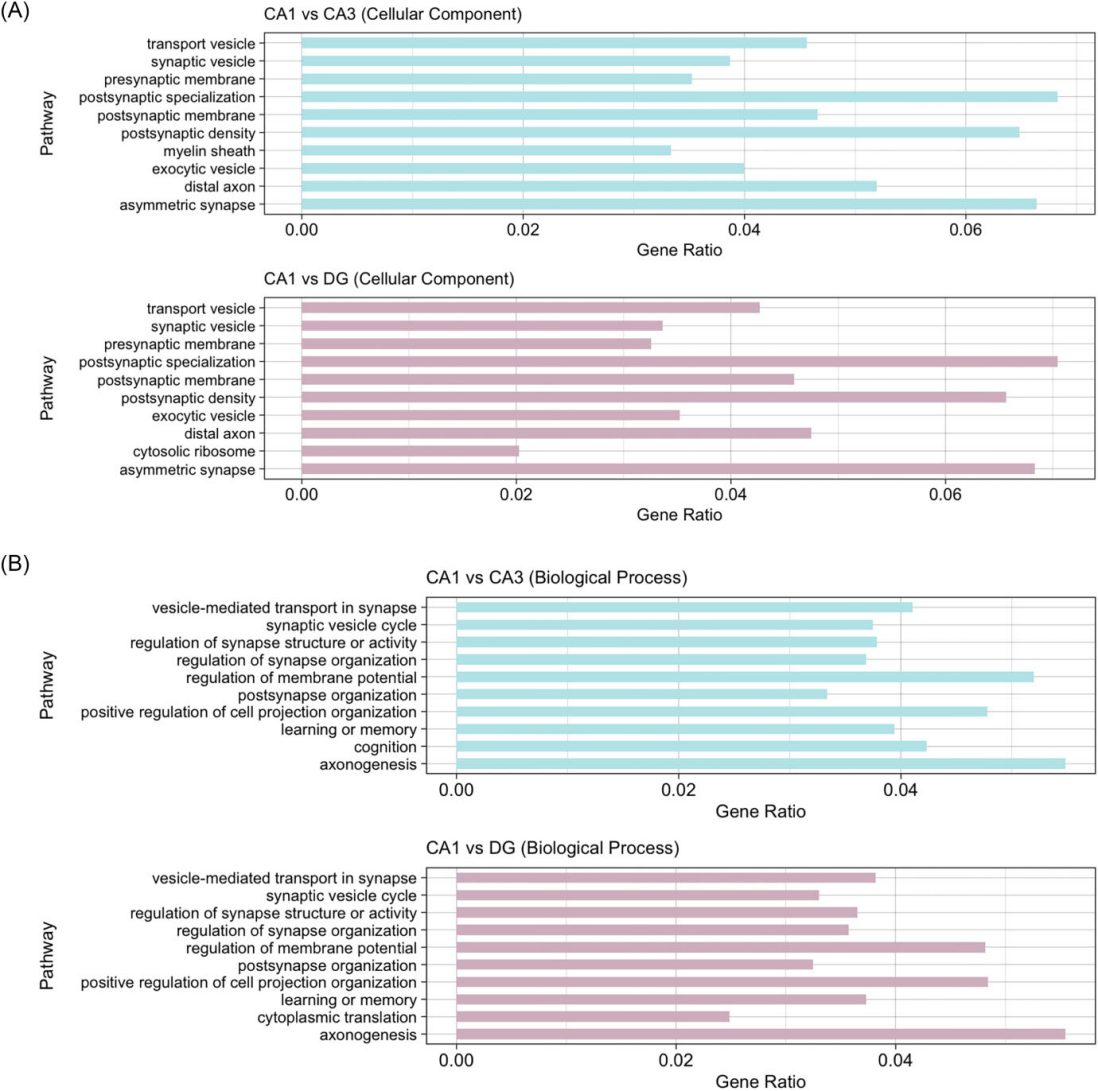
Pathway enrichment analyses of DEGs between hippocampal subfields. Enriched pathways were ranked in descending order by adjusted *P*-value and the highest-ranked 10 pathways from each category were shown. (a) Ten most significantly enriched cellular component terms from DEG sets between the CA1 and CA3, and the CA1 and DG. (b) Ten most significantly enriched biological process terms from DEG sets between the CA1 and CA3, and the CA1 and DG.

The increased abundance of Zn and expression of zinc-regulatory transcripts in the CA3 and DG likely owes to the high density of Zn-containing synapses localized in these subfields. Indeed, pathway enrichment analyses generally reflected the abundance of synaptic terminals in the CA3 and DG (Fig. 5). Analysis of GO cellular component terms highlighted pre- and post-synaptic architecture among the enriched gene sets (Fig. 5a), and Biological Process terms were particularly enriched for synaptic organization and activity, as well as axonogenesis (Fig. 5b).

### Copper enrichment in the ventricle wall

**Figure 6.**
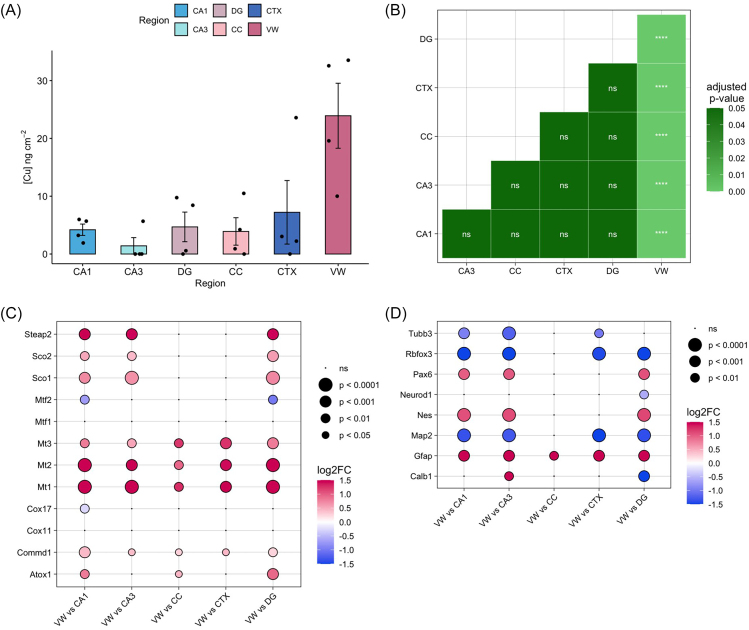
Copper abundance and expression of associated transcripts in the brain: (a) elemental concentration of copper ± SE in the CA1, CA3, DG, CC, CTX, and VW. (b) Significance of pairwise comparisons by Tukey's post-hoc test are shown between regions. Differential expression of genes relating to copper homeostasis in the VW. (c, d) Differential expression in VW relative to other subregions analysed. CA1 = cornu ammonis sector 1, CA3 = cornu ammonis sector 3, DG = dentate gyrus, CTX = cortex, CC = corpus callosum, VW = ventricle wall. *P < .05; **P < .01; ***P < .001; ^****^P < .0001.

Comparative analyses of brain subregions performed here by XFM indicated relatively low levels of copper in the CA1, CA3, DG, CC, and CTX, nearing the limit of detection, but significant enrichment of copper in the VW (Fig. 6a). These finds are concordant with previous elemental mapping studies showing a high concentration of copper at the lateral ventricles in healthy, wild-type adult mice but not within the hippocampus [13, 14, 16], where copper is believed to regulate neurogenesis [23].

The comparative transcriptome of the VW was next explored to analyse a molecular basis for copper enrichment. We were unable to detect the putative copper transporters *Ctr1, Atp7a*, and *Atp7b*, or the copper chaperone protein encoded by *Ccs*, and thus we were unable to compare their expression between subregions. However, we found the VW had significantly higher levels of several transcripts involved in cellular copper homeostasis, including *Commd1*, encoding the COMMD1 copper-induced chaperone which binds the copper transporter ATP7B [44], and *Atox1*, encoding the ATOX1 copper chaperone which delivers cytosolic copper to both ATP7A and ATP7B (Fig. 6b).

*Mt1, Mt2*, and *Mt3*, encoding metallothioneins I, II, and III, respectively, were significantly upregulated in the VW compared to all other subregions (Fig. 6b). Metallothioneins increase cellular resistance to high copper levels through sequestration of copper ions and regulation of copper transporter expression [45]. The high relative levels of metallothionein expression observed here support a key role in maintaining the physiological abundance of copper at the VW/SVZ. These findings are in strong agreement with prior X-ray absorption near edge structure (XANES) spectroscopic analysis, which indicated Cu speciation along the SVZ is consistent with metallothionein-bound Cu [46, 14]. Glial cells (astrocytes) are reported to predominantly express *Mt1* and *Mt2*, while neurons display greater expression of *Mt3*, however glial cells can express *Mt3* [47]. Our results are consistent with these relative expression profiles of *Mt*s, with greatest levels of *Mt* expression in the VW observed for *Mt1* and *Mt2*. The increased expression of *Mt3* in the VW in this study further supports the ability of glial cells to express Mt3 [47], and is consistent with studies showing *Mt3* expression in the SVZ [48]. *Sod1*, which encodes the copper/zinc superoxide dismutase (SOD1) protein essential for copper homeostasis [49], also showed consistent upregulation in the VW compared to all other subregions analysed.

*Sco1, Sco3*, and *Steap2* were upregulated in the VW when compared to all three hippocampal subfields but unchanged in comparison to the CC and CTX (Fig. 6b). *Sco1* and *Sco2* encode proteins involved in mitochondrial copper metabolism [50, 51], while *Steap2* encodes the STEAP2 metalloreductase which stimulates cellular copper uptake [52].

Markers of neurogenesis were also investigated, and exhibited strong and consistent trends in their relative expression between subregions. Both *Nes*, encoding the intermediate filament protein nestin, and *Pax6*, encoding the transcription factor PAX6, a key regulator of neurogenesis, were significantly upregulated in the VW compared to all three hippocampal subfields, but not significantly different in comparison to the CC and CTX (Fig. 6c). Although both the lateral ventricles and the hippocampus (specifically the DG) constitute the predominant neurogenic niches of the developed brain [53], our results suggest differential neurogenic activity between these regions at the time point studied (7 months), warranting further investigation across additional timepoints to see if differences are age-related. Another potential reason for the differences in neurogenesis markers observed between the VW and DG may be the selection of ROIs, which was not informed by markers of neurogenesis. More specific ROI selection, using markers of neurogenesis may enable more specific ROIs to be drawn, specifically within the DG, enabling better sensitivity and specificity to gene expression relating to neurogenesis in that region.

## Conclusions

Here, a molecular basis for metallomic heterogeneity across key subregions of the brain, specifically the hippocampal subfields and surrounding regions, was investigated *in situ* in tissue sections for the first time. Using a multimodal approach combining direct elemental mapping through XFM and spatial transcriptomic analysis of serial sections from the same brain samples, significant differences in iron, zinc, and copper levels and associated transcripts were detected across key regions of the brain. A major finding of this study was the demonstration of iron enrichment in the CA1 hippocampal subfield, concordant with a transcriptomic signature broadly reflective of higher iron regulatory activity. In particular, increased expression of multiple transcripts encoding iron chaperone proteins, IRPs, and proteins involved in intracellular iron metabolism, were observed. Likewise, for other hippocampal subfields, transcriptomics provided molecular support for the observed localized metal enrichment, such as zinc enrichment of the CA3 and DG subfields, which was concomitant with increased expression of zinc transporters ZIP7, ZnT9, ZnT10 (CA3), and ZIP6 and ZnT3 (DG). Similarly, the VW adjacent to the hippocampus is known to be enriched in copper, as also observed in this study, and transcriptomics revealed an abundance of copper-binding proteins (specifically the MTs, MT1-3) in this region. A limitation of this study however, was the failure to detect *Ctr1, Cp, Atp7a*, and *Atp7b*, which code for other key copper proteins, as well as the iron exporter, *Slc40a1*. We believe that the failure to detect the above-mentioned genes does not indicate a true ‘absence’ but rather more likely the RNA had degraded to levels below the minimum detection limits. It is hoped further work can continue to optimise sample preparation and workflows such that these genes can be detected in the future.

Another limitation of this study is that neither XFM nor transcriptomics reveal metal ion speciation. Further integration of additional techniques such as XANES spectroscopy, or fluorescent sensors of metal ions that are compatible with tissue sections, may provide invaluable information on the relationships between the labile and protein-bound metal pools and their regulation by gene expression.

In summary, this study provides an important template for others to integrate transcriptomics into multimodal workflows investigating the neurometallome of the brain, as well as other tissue types. Ultimately, increased understanding of the transcriptomic basis of the metallome within specific brain cells, and how this changes in pathological states, will be vital for understanding the role of metal ions in brain function, brain disease, or brain damage, and may reveal new lines of therapeutic or restorative intervention. In particular, there is substantial interest in the role of iron in brain function and malfunction during natural ageing and neurodegeneration (driven by either injury or disease), and the correlative workflow we have outlined provides a new analytical pipeline through which brain iron homeostasis may be investigated.

## Supplementary Material

mfaf009_Supplemental_File

## Data Availability

The data underlying this article will be shared on reasonable request to the corresponding author.

## References

[1] Hackett MJ, Hollings A, Caine S *et al*. Elemental characterisation of the pyramidal neuron layer within the rat and mouse hippocampus. Metallomics. 2019;11:151–65. 10.1039/c8mt00230d 30398510

[2] Ellison G, Duong L, Hollings A *et al*. Characterising murine hippocampal iron homeostasis, in relation to markers of brain inflammation and metabolism, during ageing. Metallomics. 2022;14:p.mfac064. 10.1093/mtomcs/mfac064 36066906

[3] Jorgenson LA, Sun M, O'Connor M *et al*. Fetal iron deficiency disrupts the maturation of synaptic function and efficacy in area CA1 of the developing rat hippocampus. Hippocampus. 2005;15:1094–102. 10.1002/hipo.20128 16187331

[4] Yehuda S, Youdim ME, Mostofsky DI. Brain iron-deficiency causes reduced learning capacity in rats. Pharmacol Biochem Behav. 1986;25:141–4. 10.1016/0091-3057(86)90244-3 3018790

[5] Agrawal S, Berggren KL, Marks E *et al*. Impact of high iron intake on cognition and neurodegeneration in humans and in animal models: a systematic review. Nutr Rev. 2017;75:456–70. 10.1093/nutrit/nux015 28505363 PMC5914328

[6] Levi S, Ripamonti M, Moro AS *et al*. Iron imbalance in neurodegeneration. Mol Psychiatry. 2024;29:1139–52. 10.1038/s41380-023-02399-z. Epub 2024 Jan 12. 38212377 PMC11176077

[7] Raven EP, Lu PH, Tishler TA *et al*. Increased iron levels and decreased tissue integrity in hippocampus of Alzheimer's disease detected in vivo with magnetic resonance imaging. JAD. 2013;37:127–36. 10.3233/JAD-130209 23792695

[8] Hackett MJ. A commentary on studies of brain iron accumulation during ageing. J Biol Inorg Chem. 2024;29:385–94. 10.1007/s00775-024-02060-2 38735007 PMC11186910

[9] Andreini C, Bertini I, Rosato A. Metalloproteomes: a bioinformatic approach. Acc Chem Res. 2009;42:1471–9. 10.1021/ar900015x 19697929

[10] Tóth K. Zinc in neurotransmission. Annu Rev Nutr. 2011;31:139–53. 10.1146/annurev-nutr-072610-145218 21548772

[11] Watt NT, Whitehouse IJ, Hooper NM. The role of zinc in Alzheimer's disease. Int J Alzheimer's Dis. 2011;2011:971021. 10.4061/2011/971021 PMC3010690 21197404

[12] Liu LL, van Rijn RM, Zheng W. Copper Modulates Adult Neurogenesis in Brain Subventricular Zone. Int J Mol Sci. 2022;23:9888. 10.3390/ijms23179888 36077284 PMC9456150

[13] Fu S, Jiang W, Zheng W. Age-dependent increase of brain copper levels and expressions of copper regulatory proteins in the subventricular zone and choroid plexus. Front Mol Neurosci. 2015;8:22. 10.3389/fnmol.2015.00022 26106293 PMC4458609

[14] Pushkar Y, Robison G, Sullivan B *et al*. Aging results in copper accumulations in glial fibrillary acidic protein-positive cells in the subventricular zone. Aging Cell. 2013;12:823–32. 10.1111/acel.12112. Epub 2013 Jul 8. 23738916 PMC3772960

[15] Robison G, Zakharova T, Fu S *et al*. X-ray fluorescence imaging of the hippocampal formation after manganese exposure. Metallomics. 2013;5:1554–65. 10.1039/c3mt00133d 23999853 PMC3892963

[16] Sullivan B, Robison G, Pushkar Y *et al*. Copper accumulation in rodent brain astrocytes: a species difference. J Trace Elem Med Biol. 2017;39:6–13. 10.1016/j.jtemb.2016.06.011. Epub 2016 Jul 6. 27908425 PMC5141684

[17] Ulgen E, Ozisik O, Sezerman OU. pathfindR: an R package for comprehensive identification of enriched pathways in omics data through active subnetworks. Front Genet. 2019;10:858. Doi: 10.3389/fgene.2019.00858 31608109 PMC6773876

[18] Jassal B, Matthews L, Viteri G *et al*. The reactome pathway knowledgebase. Nucleic Acids Res. 2020;48:D498–503. 10.1093/nar/gkz1031 31691815 PMC7145712

[19] Howard DL, de Jonge MD, Afshar N *et al*. The XFM beamline at the Australian synchrotron. J Synchrotron Rad. 2020;27: 1447–58. 10.1107/S1600577520010152 32876622

[20] Brunette KE, Tran PV, Wobken JD *et al*. Gestational and neonatal iron deficiency alters apical dendrite structure of CA1 pyramidal neurons in adult rat hippocampus. Dev Neurosci. 2010;32:238–48. 10.1159/000314341. Epub 2010 Aug 6. 20689287 PMC3214841

[21] Singh A, Mohan ML, Isaac AO *et al*. Prion protein modulates cellular iron uptake: a novel function with implications for prion disease pathogenesis. PLoS One. 2004;4:e4468. 10.1371/journal.pone.0004468. Epub 2009 Feb 12. Erratum in: PLoS ONE. 2009;4(2). PMC2637434 19212444

[22] Singh A, Kong Q, Luo X *et al*. Prion protein (PrP) knock-out mice show altered iron metabolism: a functional role for PrP in iron uptake and transport. PLoS One. 2009;4:e6115. 10.1371/journal.pone.0006115 19568430 PMC2699477

[23] Pushie MJ, Pickering IJ, Martin GR *et al*. Prion protein expression level alters regional copper, iron and zinc content in the mouse brain. Metallomics. 2011;3:206–14. 10.1039/c0mt00037j 21264406

[24] Cheli VT, Santiago González DA, Marziali LN *et al*. The divalent metal transporter 1 (DMT1) is required for iron uptake and normal development of oligodendrocyte progenitor cells. J. Neurosci. 2018;38:9142–59. 10.1523/JNEUROSCI.1447-18.2018. Epub 2018 Sep 6. 30190412 PMC6199407

[25] Graham RM, Chua AC, Herbison CE *et al*. Liver iron transport. World J Gastroenterol. 2007;13:4725. 10.3748/wjg.v13.i35.4725 17729394 PMC4611194

[26] Garrick MD, Singleton ST, Vargas F *et al*. DMT1: which metals does it transport?. Biol Res. 2006;39:79–85. 10.4067/s0716-97602006000100009 16629167

[27] Shi H, Bencze KZ, Stemmler TL et al. A cytosolic iron chaperone that delivers iron to ferritin. Science. 2008;320:1207–10. 10.1126/science.1157643 18511687 PMC2505357

[28] Philpott CC, Jadhav S. The ins and outs of iron: Escorting iron through the mammalian cytosol. Free Radical Biol Med. 2019;133:112–7. 10.1016/j.freeradbiomed.2018.10.411. Epub 2018 Oct 12. 30321701

[29] Moroishi T, Nishiyama M, Takeda Y *et al*. The FBXL5-IRP2 axis is integral to control of iron metabolism in vivo. Cell Metab. 2011;14:339–51. 10.1016/j.cmet.2011.07.011 21907140

[30] Moroishi T, Yamauchi T, Nishiyama M *et al*. HERC2 targets the iron regulator FBXL5 for degradation and modulates iron metabolism. J Biol Chem. 2014;289:16430–41. 10.1074/jbc.M113.541490. Epub 2014 Apr 28. 24778179 PMC4047410

[31] Meyron-Holtz EG, Ghosh MC, Iwai K *et al*. Genetic ablations of iron regulatory proteins 1 and 2 reveal why iron regulatory protein 2 dominates iron homeostasis. EMBO J. 2004;23:386–95. 10.1038/sj.emboj.7600041. Epub 2004 Jan 15. 14726953 PMC1271751

[32] Reinert A, Morawski M, Seeger J *et al*. Iron concentrations in neurons and glial cells with estimates on ferritin concentrations. BMC Neurosci. 2019;20:1–4. 10.1186/s12868-019-0507-7 31142282 PMC6542065

[33] Healy S, McMahon J, Owens P *et al*. Significant glial alterations in response to iron loading in a novel organotypic hippocampal slice culture model. Sci Rep. 2016;6:36410. 10.1038/srep36410 27808258 PMC5093415

[34] Teng L, Nakada M, Zhao SG *et al*. Silencing of ferrochelatase enhances 5-aminolevulinic acid-based fluorescence and photodynamic therapy efficacy. Br J Cancer. 2011;104:798–807. 10.1038/bjc.2011.12. Epub 2011 Feb 8. 21304523 PMC3048207

[35] Frederickson CJ, Danscher G. Zinc-containing neurons in hippocampus and related CNS structures. Prog Brain Res. 1990;83:71–84. 10.1016/s0079-6123(08)61242-x 2203108

[36] Pushie MJ, Hollings A, Reinhardt J *et al*. Sample preparation with sucrose cryoprotection dramatically alters Zn distribution in the rodent hippocampus, as revealed by elemental mapping. J Anal At Spectrom. 2020;35:2498–508. 10.1039/d0ja00323a. Epub 2020 Aug 19. 33795908 PMC8009441

[37] Cassell MD, Brown MW. The distribution of Timm's stain in the nonsulphide-perfused human hippocampal formation. J Compar Neurol. 1984;222:461–71. 10.1002/cne.902220311 6199383

[38] De Benedictis CA, Haffke C, Hagmeyer S *et al*. Expression analysis of zinc transporters in nervous tissue cells reveals neuronal and synaptic localization of ZIP4. Int J Mol Sci. 2021;22:4511. 10.3390/ijms22094511 33925953 PMC8123391

[39] Woodruff G, Bouwkamp CG, de Vrij FM *et al*. The zinc transporter SLC39A7 (ZIP7) is essential for regulation of cytosolic zinc levels. Mol Pharmacol. 2018;94:1092–100. 10.1124/mol.118.112557. Epub 2018 Jul 6. 29980658

[40] Perez Y, Shorer Z, Liani-Leibson K *et al*. SLC30A9 mutation affecting intracellular zinc homeostasis causes a novel cerebro-renal syndrome. Brain. 2017;140:928–39. 10.1093/brain/awx013 28334855 PMC5837213

[41] Lichten LA, Ryu MS, Guo L *et al*. MTF-1-mediated repression of the zinc transporter Zip10 is alleviated by zinc restriction. PLoS One. 2011;6:e21526. 10.1371/journal.pone.0021526. Epub 2011 Jun 27. 21738690 PMC3124522

[42] Jeong J, Eide DJ. The SLC39 family of zinc transporters. Mol Aspects Med. 2013;34:612–9. 10.1016/j.mam.2012.05.011 23506894 PMC3602797

[43] Huang L, Tepaamorndech S. The SLC30 family of zinc transporters—a review of current understanding of their biological and pathophysiological roles. Mol Aspects Med. 2013;34:548–60. 10.1016/j.mam.2012.05.008 23506888

[44] Weiskirchen R, Penning LC. COMMD1, a multi-potent intracellular protein involved in copper homeostasis, protein trafficking, inflammation, and cancer. J Trace Elem Med Biol. 2021;65:126712. 10.1016/j.jtemb.2021.126712. Epub 2021 Jan 7. 33482423

[45] Tapia L, González-Agüero M, Cisternas MF *et al*. Metallothionein is crucial for safe intracellular copper storage and cell survival at normal and supra-physiological exposure levels. Biochem J. 2004;378:617–24. 10.1042/BJ20031174 14627437 PMC1223976

[46] Sullivan B, Robison G, Osborn J *et al*. On the nature of the Cu-rich aggregates in brain astrocytes. Redox Biol. 2017;11:231–9. 10.1016/j.redox.2016.12.007. Epub 2016 Dec 9. 28012438 PMC5198742

[47] Lee SJ, Koh JY. Roles of zinc and metallothionein-3 in oxidative stress-induced lysosomal dysfunction, cell death, and autophagy in neurons and astrocytes. Mol Brain. 2010;3:1–9. 10.1186/1756-6606-3-30 20974010 PMC2988061

[48] MacDonald A, Lu B, Caron M *et al*. Single cell transcriptomics of ependymal cells across age, region and species reveals cilia-related and metal ion regulatory roles as major conserved ependymal cell functions. Front Cell Neurosci. 2021;15:703951. 10.3389/fncel.2021.703951 34335193 PMC8319996

[49] Bourassa MW, Brown HH, Borchelt DR *et al*. Metal-deficient aggregates and diminished copper found in cells expressing SOD1 mutations that cause ALS. Front Aging Neurosci. 2014;6:110. 10.3389/fnagi.2014.00110 24982630 PMC4059277

[50] Horng YC, Leary SC, Cobine PA *et al*. Human Sco1 and Sco2 function as copper-binding proteins. J Biol Chem. 2005;280:34113–22. 10.1074/jbc.M506801200. Epub 2005 Aug 9. Erratum in: J Biol Chem. 2005 Dec 9;280(49):41122. 16091356

[51] Jaksch M, Paret C, Stucka R *et al*. Cytochrome c oxidase deficiency due to mutations in SCO2, encoding a mitochondrial copper-binding protein, is rescued by copper in human myoblasts. Hum Mol Genet. 2001;10:3025–35. 10.1093/hmg/10.26.3025 11751685

[52] Ohgami RS, Campagna DR, McDonald A *et al*. The Steap proteins are metalloreductases. Blood. 2006;108:1388–94. 10.1182/blood-2006-02-003681. Epub 2006 Apr 11. 16609065 PMC1785011

[53] Jurkowski MP, Bettio L, K Woo E *et al*. Beyond the hippocampus and the SVZ: adult neurogenesis throughout the brain. Front Cell Neurosci. 2020;14:576444. 10.3389/fncel.2020.576444 33132848 PMC7550688

