# Supplementary material for: Correlative analysis of metallomic gene expression and metal ion content within the mouse hippocampus
Source: Metallomics. 2025 Apr 2;17(4):mfaf009. doi: 10.1093/mtomcs/mfaf009 (PMC12086694; doi:10.1093/mtomcs/mfaf009)
Supplement: mfaf009_Supplemental_File [file mfaf009_Supplemental_File.docx]

**Supplementary Information**

**Correlative analysis of metallomic gene expression and metal ion content within the mouse hippocampus**

Somayra S. A. Mamsa,^1,2^ Gaewyn Ellison,^1,3^ Julia Koehn,^1^ Keea Inder-Smith,^1,4^ Cameron W. Evans,^2^ Ross M. Graham,^1,4^ Daryl L. Howard,^5^ Mark J. Hackett^1,3*^

**Supplementary Table 1**

| **Group 1** | **Group 2** | **Null Value** | **Estimate** | **Confidence Interval** | **Adjusted p-value** | **Significance Level** |
| --- | --- | --- | --- | --- | --- | --- |
| CA1 | CA3 | 0 | -18.04875 | -29.069787 – -7.027713 | 5.22e-04 | *** |
| CA1 | CC | 0 | -26.48325 | -37.50428 – -15.462213 | 2.39e-06 | **** |
| CA1 | CTX | 0 | -24.21550 | -35.23653 – -13.194463 | 9.57e-06 | **** |
| CA1 | DG | 0 | -16.73850 | -27.759537 – -5.717463 | 1.25e-03 | ** |
| CA1 | VW | 0 | -21.80275 | -32.82378 – -10.781713 | 4.43e-05 | **** |
| CA3 | CC | 0 | -8.43450 | -21.160497 – 4.291497 | 3.41e-01 | ns |
| CA3 | CTX | 0 | -6.16675 | -18.892747 – 6.559247 | 6.62e-01 | ns |
| CA3 | DG | 0 | 1.31025 | -11.415747 – 14.036247 | 9.99e-01 | ns |
| CA3 | VW | 0 | -3.75400 | -16.479997 – 8.971997 | 9.37e-01 | ns |
| CC | CTX | 0 | 2.26775 | -10.458247 – 14.993747 | 9.93e-01 | ns |
| CC | DG | 0 | 9.74475 | -2.981247 – 22.470747 | 2.04e-01 | ns |
| CC | VW | 0 | 4.68050 | -8.045497 – 17.406497 | 8.57e-01 | ns |
| CTX | DG | 0 | 7.47700 | -5.248997 – 20.202997 | 4.68e-01 | ns |
| CTX | VW | 0 | 2.41275 | -10.313247 – 15.138747 | 9.91e-01 | ns |
| DG | VW | 0 | -5.06425 | -17.790247 – 7.661747 | 8.13e-01 | ns |

***Results of Tukey’s post-hoc testing for iron levels.***

**Supplementary Table 2**

| **Group 1** | **Group 2** | **Null Value** | **Estimate** | **Confidence Interval** | **Adjusted p-value** | **Significance Level** |
| --- | --- | --- | --- | --- | --- | --- |
| CA1 | CA3 | 0 | 81.76700 | 64.05374 – 99.48026 | 1.62e-11 | **** |
| CA1 | CC | 0 | -30.32225 | -48.03551 – -12.60899 | 3.03e-04 | *** |
| CA1 | CTX | 0 | -11.71275 | -29.42601 – 6.00051 | 3.43e-01 | ns |
| CA1 | DG | 0 | 131.31250 | 113.59924 – 149.02576 | 2.42e-14 | **** |
| CA1 | VW | 0 | -10.93225 | -28.64551 – 6.78101 | 4.15e-01 | ns |
| CA3 | CC | 0 | -112.08925 | -132.54276 – -91.63574 | 5.52e-13 | **** |
| CA3 | CTX | 0 | -93.47975 | -113.93326 – -73.02624 | 1.98e-11 | **** |
| CA3 | DG | 0 | 49.54550 | 29.09199 – 69.99901 | 2.10e-06 | **** |
| CA3 | VW | 0 | -92.69925 | -113.15276 – -72.24574 | 2.33e-11 | **** |
| CC | CTX | 0 | 18.60950 | -1.84401 – 39.06301 | 8.88e-02 | ns |
| CC | DG | 0 | 161.63475 | 141.18124 – 182.08826 | 2.38e-14 | **** |
| CC | VW | 0 | 19.39000 | -1.06351 – 39.84351 | 6.99e-02 | ns |
| CTX | DG | 0 | 143.02525 | 122.57174 – 163.47876 | 2.60e-14 | **** |
| CTX | VW | 0 | 0.78050 | -19.67301 – 21.23401 | 1.00e+00 | ns |
| DG | VW | 0 | -142.24475 | -162.69826 – -121.79124 | 2.64e-14 | **** |

***Results of Tukey’s post-hoc testing for zinc levels.***

**Supplementary Table 3**

| **Group 1** | **Group 2** | **Null Value** | **Estimate** | **Confidence Interval** | **Adjusted p-value** | **Significance Level** |
| --- | --- | --- | --- | --- | --- | --- |
| CA1 | CA3 | 0 | -2.77950 | -10.887127 – 5.328127 | 8.89e-01 | ns |
| CA1 | CC | 0 | -0.69150 | -8.799127 – 7.416127 | 1.00e+00 | ns |
| CA1 | CTX | 0 | -0.75700 | -8.864627 – 7.350627 | 1.00e+00 | ns |
| CA1 | DG | 0 | 0.49375 | -7.613877 – 8.601377 | 1.00e+00 | ns |
| CA1 | VW | 0 | 24.93325 | 16.825623 – 33.040877 | 3.66e-08 | **** |
| CA3 | CC | 0 | 2.08800 | -7.273882 – 11.449882 | 9.81e-01 | ns |
| CA3 | CTX | 0 | 2.02250 | -7.339382 – 11.384382 | 9.83e-01 | ns |
| CA3 | DG | 0 | 3.27325 | -6.088632 – 12.635132 | 8.80e-01 | ns |
| CA3 | VW | 0 | 27.71275 | 18.350868– 37.074632 | 7.19e-08 | **** |
| CC | CTX | 0 | -0.06550 | -9.427382 – 9.296382 | 1.00e+00 | ns |
| CC | DG | 0 | 1.18525 | -8.176632 – 10.547132 | 9.99e-01 | ns |
| CC | VW | 0 | 25.62475 | 16.262868 – 34.986632 | 2.79e-07 | **** |
| CTX | DG | 0 | 1.25075 | -8.111132 – 10.612632 | 9.98e-01 | ns |
| CTX | VW | 0 | 25.69025 | 16.328368 – 35.052132 | 2.67e-07 | **** |
| DG | VW | 0 | 24.43950 | 15.077618 – 33.801382 | 6.19e-07 | **** |

***Results of Tukey’s post-hoc testing for copper levels.***


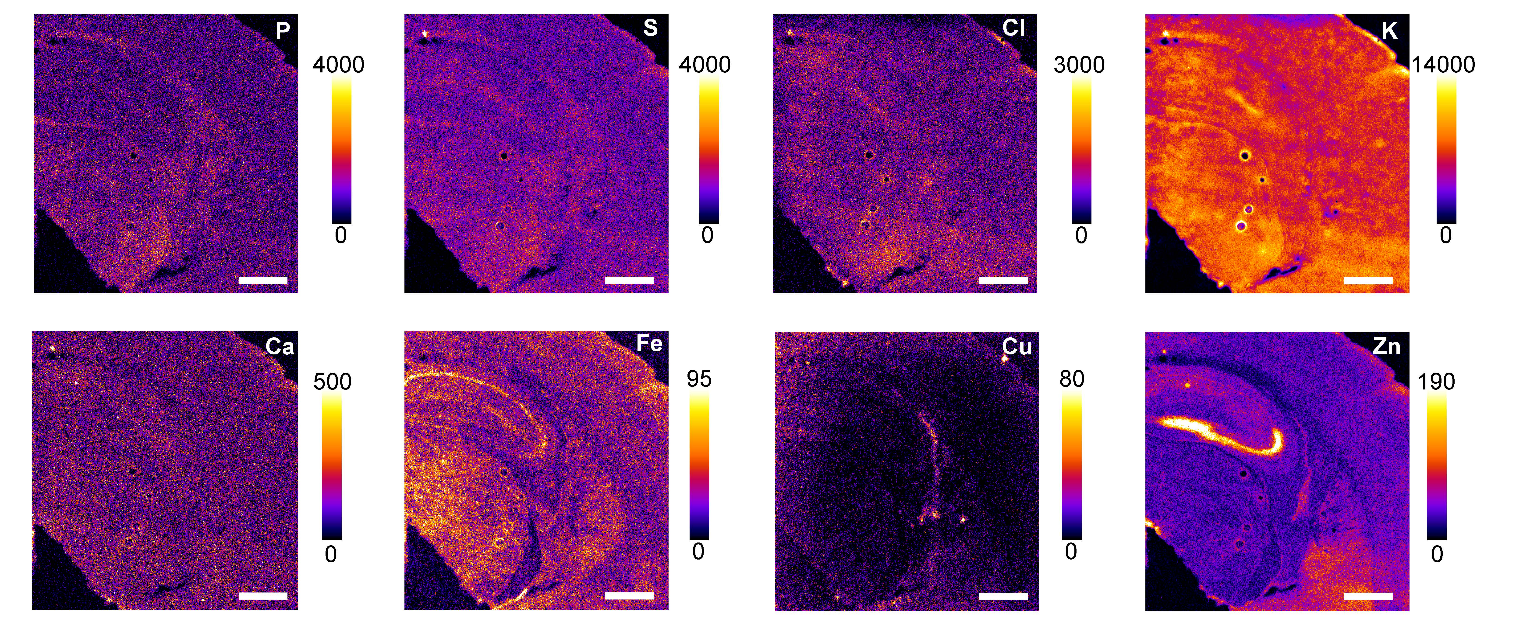


***Supplementary Figure SI1****. Elemental maps of all elements detected at 0.1 ms dwell time. Scale bar = 500 µm. Units for areal density are ng cm^-2^.*
